# Use of an Interactive, Faith-Based Kiosk by Congregants of Four Predominantly, African–American Churches in a Metropolitan Area

**DOI:** 10.3389/fpubh.2014.00106

**Published:** 2014-08-05

**Authors:** Scott A. Dulchavsky, Wilma J. Ruffin, Dayna A. Johnson, Chad Cogan, Christine L. M. Joseph

**Affiliations:** ^1^Department of Surgery, Henry Ford Hospital, Detroit, MI, USA; ^2^Department of Public Health Sciences, Henry Ford Hospital, Detroit, MI, USA

**Keywords:** kiosk, faith-based interventions, health information, health ministry, health disparities, chronic disease, minority health

## Abstract

Chronic diseases are prevalent in ethnic communities. Churches represent a potent resource for targeted health promotion. A faith-based kiosk was developed as an informational tool and placed in four predominantly (>80%) African-American churches. Congregants were surveyed to describe kiosk-use, kiosk-user characteristics, health status, and self-reported behavior changes attributed to the kiosk. We analyzed 1,573 questionnaires. Mean age of respondents was 46.4 years and >70% were women. “Older” congregations (mean age ≥46.1 years) had more reports of diabetes (*p* = 0.002) and heart diseases (*p* = 0.01) than younger churches (mean age ≤44.1), whereas asthma was more prevalent in the latter (*p* < 0.001). Prevalence of obesity (40%) was similar across churches (*p* = 0.570). Kiosk-use was reported by 420 (26.7%) respondents. Compared to non-users, kiosk-users were >40 years (*p* < 0.001), and reported >two health conditions, adjusted Odds Ratio (95% Confidence Interval) = 1.43 (1.0–2.0), *p* = 0.05. Male kiosk-users preferred to select disease-specific content, aOR = 1.87 (1.10–3.17), *p* = 0.02, while females tended to select information about supportive community resources, aOR = 0.49 (0.23–1.04), *p* = 0.062. Knowledge of kiosk-user characteristics and the “health status” of a congregation, provide an opportunity for targeted, church-based health promotion.

## Introduction

Discrimination and lack of health care resources in vulnerable communities contribute to unequal health outcomes. Access to health information and care is a great challenge for underserved populations in the United States and abroad, as low literacy may compound limited access to helpful health information (1, 2). These barriers to health information are difficult to overcome with traditional strategies.

Churches remain a consistent source of strength in ethnic and poorer communities, providing a home for the individual and the collective physical, emotional, and spiritual health of communities (3). Indeed, many believe that the church can play an important role in meeting the health needs of a congregation (4, 5).

The use of emerging information technology to provide health information on major urban health problems has the promise to empower individuals in underserved communities to improve their own health. Multimedia instructional methods such as video or pictorial-based stories have been shown to be substantially more effective than text-based solutions in a variety of communities, including underserved and ethnic populations (6–11). The effect of enhanced retention of information with visual learning is particularly pronounced in geriatric or low literacy groups, which comprise a large percentage of Medicare recipients (12).

The end of the twentieth century witnessed the growth of using kiosks to provide non-text-based personal communication on health issues. Gielen, et al. (13) demonstrated that parents in an urban pediatric hospital emergency department serving a predominantly low-income population increased their knowledge and attitudes about public health-related information after using kiosks based in this location (13). In one survey of kiosk-users, the majority responded that they planned to apply what they read and almost 50% planned to speak with their physician about what they read on the kiosk (14). A significant aspect of community-based kiosks is that they can provide tailored information to users. Research indicates that tailored information is more likely than non-tailored information to be viewed as personally relevant, and is more likely to be read and acted upon (15).

Henry Ford Health System (HFHS) partnered with the existing community infrastructures to improve health care literacy through a church-based health information kiosk system. This faith-based medical kiosk was designed with an intuitive, touch screen interface, and targeted populations that may have low health literacy skills. Each free-standing kiosk is customized to the congregation and incorporates messaging from church leaders. Users enter simple demographic information to direct each interaction. The touch screen kiosks are simple to use, requiring no computer knowledge and are built at an appropriate literacy level for the anticipated users.

We placed faith-based kiosks in four large, urban churches, under the guidance of the church health ministry. We then administered a questionnaire to church congregants 1–2 years after kiosk installation. The objective of the questionnaire was to (1) estimate kiosk-use among congregants, (2) describe kiosk-user characteristics, and health status, and (3) describe self-reported behavior changes attributed to use of the kiosk.

## Materials and Methods

### Software development

**Figure 1:**
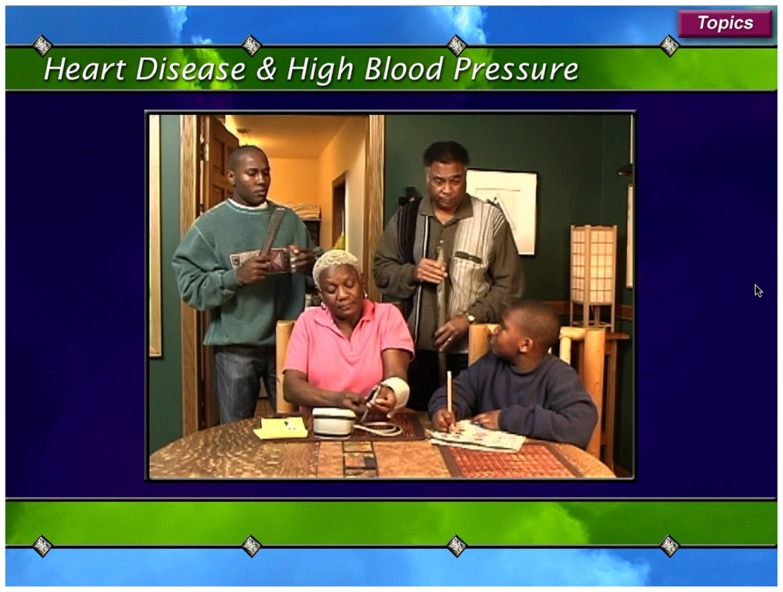
**Faith-based Kiosk: “Blood Pressure Module grandmother taking blood pressure with grandson, son and grandfather”**.

This interactive, multimedia software program was designed on an industry standard platform using a Director^TM^ development program. The kiosk provides examples of positive family interaction to provide good role models of healthy behavior. Each medical condition presented is contextualized in a family setting. A multigenerational family unit interacts candidly in a short video while discussing each condition (Figure 1).

The program begins with an introductory message by the pastor of the church explaining the purpose of the Health Kiosk, encouraging the user to explore sections of interest. Following the introductory video, questions are asked of the user to tailor the experience by age, gender, and health status. Additional questions are posed regarding smoking, exercise, and whether the user has a personal physician which set the user population baseline. The major modules included are cholesterol screening, hypertension, osteoporosis, memory loss, depression, sexually transmitted diseases, heart disease, stroke, cancer, HIV/AIDS, and healthy living. These modules provide detailed information on common symptoms, specific risk factors, preventative measures, and appropriate screening guidelines for early detection. Following the video, there is a more didactic presentation of evidence based information about the topics combined with rich media support, culturally appropriate, and diverse material. After the module, there is a second tier of interactions available – all calls to action in two major categories: Healthy Lifestyle Choices and Community support.

The software was fully developed in collaboration with Butler Graphics, Inc. The program was pilot-tested with groups from the faith-based community to assess user friendliness and design, to determine the key health issues of most concern to community members, and to determine how it might benefit the congregation. To our knowledge, there is no other comparable software on the market. HFHS has begun the application process for a patent.

### Survey of church membership

To assess the impact of the kiosk (usage rates, self-report of behavior change, and overall acceptability), a questionnaire was developed through a collaborative effort between HFHS and Church Health Ministry Leaders and Pastors. Churches also discussed how they might use the health information in the kiosks. The questionnaire had a total of 27 items. Requested was respondent demographic information, respondent experiences with the kiosk, the health status of the respondent, and the health status of respondent family members. Names of respondents or any identifying information was not requested. The questionnaire was pilot-tested in a subset of congregants and leaders from each church participant to develop a standardized protocol for administration. The HFHS IRB approved the questionnaire and the questionnaire protocol.

The questionnaire was administered to the four participating churches from March–May, 2009. Letters were sent to Pastors and Health Ministries informing them of the upcoming evaluation and requesting their support. In preparation, research staff organized an orientation/training session with church staff members to discuss the logistics of administering the survey. Churches were contacted 3 days prior to survey distribution to confirm participation. Questionnaires were distributed by research staff at each service on one Sunday and handed to every member of the church that was 18 years of age or older upon their entry into the sanctuary. During and following the service, the congregants were reminded to complete and submit their questionnaire to an HFHS staff member in a designated area. Each church received $5.00 for every completed survey, with the maximum allowance of $5,000. As congregants submitted their questionnaire, they were given a tote bag, key chain, first aid kit, or small key chain flashlight.

### Statistical analysis

Demographic information and self-reported prevalence of health conditions was summarized for each church. Age was reported using means and standard deviations. To examine differences across churches, a one-way analysis of variance was used to test for statistically significant differences in mean age for at least one comparison among the four churches. A chi-square test was used to determine if responses were statistically different across the four churches. Associations with a *p*-value <0.05 were considered statistically significant.

To assess the independent association of demographic variables and other survey items to use of the kiosk, a multiple logistic regression model with user/non-user as the dependent variable, was constructed and included all variables of interest (age, race, gender, number of health conditions reported, and family member with a health condition), adjusting for church membership. In these models, prevalence of health condition(s) was coded as none, 1, 2, or 3 or more.

We also examined the relationship between gender and section viewed. Only sections with bivariate chi-square results yielding a *p*-value <0.20 were examined further using multiple logistic regression. For these sections, a multiple logistic regression was run in which section viewed (yes/no) as the dependent variable. Each model contained gender, age, and church membership.

Finally, we created a model to assess factors associated with self-report of behavior change. Variables in the model were age, race, gender, self-report of health condition(s) (coded as none, 1, 2, or 3 or more), having a family member with a health condition (yes/no). The model was also adjusted for church membership.

As noted above, less stringent *p*-values (<0.20) were used in the building of some models, however, for the final multiple logistic regression models, *p* < 0.05 was considered statistically significant. All analyses were conducted using SAS software version 9.2.

## Results

### Characteristics of respondents and estimated use of kiosk

The four participating churches ranged in congregation membership from 1500 to 6000, with usual church attendance on Sunday ranging from 500 to 2000 according to estimates provided by church staff. The estimated percentage of surveys returned was 421/500 (84%), 38% (*n* = 305/800) 40% (*n* = 792/2000), and 46% (*n* = 461/1000) for a combined total of 1979 for the four participating churches (Church A, B, C, D, respectively), of which 79.5% (*n* = 1573) had data usable for this descriptive analysis. Of those, 420 (26.7%) reported using the kiosk (range 19.1–35.4%).

Respondent characteristics are presented in Table 1, along with demographics and self-report of respondent and respondent family members’ health status. Respondents from all four churches were mostly African-American (>80%) and the percentage of males ranged from 18.1 to 27.5%. Across churches, the range in mean age was 41.6–54.8 (overall *p* < 0.001). The age difference between the church with the oldest surveyed members (Church A) and the youngest (Church B) was statistically significant (*p* < 0.001).

**Table 1:** **Characteristics of survey respondents by Church^a^**.

			Church A	Church B	Church C	Church D	Overall *p*-value^b^
Total surveyed			421	305	792	461	–
Estimated usual church attendance			*N* = 500	*N* = 800	*N* = 2000	*N* = 1000	
Reported using kiosk			61 (19.1%)	80 (32.4%)	144 (23.0%)	135 (35.4%)	<0.01
Age, mean (SD)			54.8 (14.9)	41.6 (14.7)	44.1 (13.4)	46.1 (14.4)	<0.001^c^
African-American, *n* (%)			373 (88.6%)	258 (84.6%)	661 (83.5%)	416 (90.2%)	0.003
Male, *n* (%)			95 (26.2%)	68 (26.1%)	125 (18.1%)	118 (27.5%)	<0.001
Family member with health condition, *n* (%)			140 (50.5%)	120 (55.6%)	289 (54.3%)	153 (46.9%)	0.120

	**US estimates age adjusted percentages**		
**Health Conditions, *n* (%)**	**All adults (%)^d^**	**US African- Americans (%)^e^**					

Diabetes	8.7	13.1	81 (28.7%)	46 (23.6%)	98 (18.6%)	58 (17.4%)	0.002
Heart disease	11.5	11.2	38 (15.3%)	17 (9.9%)	42 (8.6%)	24 (7.7%)	0.013
High blood pressure	24.0	32.2	216 (65.7%)	108 (49.3%)	269 (45.9%)	156 (41.9%)	<0.001
Obesity	27.2	37.6	110 (42.6%)	74 (39.8%)	196 (37.7%)	140 (40.9%)	0.570
HIV/AIDs or STDs	<1	<1	20 (8.9%)	22 (13.9%)	61 (13.1%)	28 (9.3%)	0.165
Asthma	13.0^f^	13.8	53 (22.0%)	45 (25.4%)	98 (20.3%)	36 (11.5%)	<0.001
Arthritis	22.1	23.1	138 (48.9%)	62 (32.8%)	151 (29.0%)	87 (26.0%)	<0.001
Cancer	7.9^g^	4.4	30 (12.4%)	15 (9.3%)	40 (8.3%)	20 (6.5%)	0.106
Osteoporosis^h^	18	7.0	15 (16.9%)	5 (20.0%)	13 (13.3%)	14 (11.3%)	0.623

*^a^Percentages calculated from non-missing responses*.

*^b^All *p*-values based on Chi-squared test unless otherwise noted*.

*^c^*p*-Value based on one-way ANOVA*.

*^d^18 years of age and older*.

*^e^Includes persons selecting Black or African-American only*.

*^f^Ever diagnosed*.

*^g^Denominator is women aged 50 years and up*.

Percentage of respondents reporting certain health conditions is shown in Table 1. Church A, with the highest overall mean age of 54.8 years, also had the highest reports of diabetes, heart disease, hypertension, and arthritis. Church B, with the lowest overall mean age of 41.6 years, was only highest in self-report of asthma. Despite significant age differences in the congregants surveyed, the percentage of respondents reporting obesity across churches was similar. Church B, with the youngest mean age for those surveyed, had a self-reported percentage of obesity similar to that of churches with higher overall mean ages, 39.8 vs. 42.6% and 40.9% for Churches B, A, and D, respectively (Table 1). With the exception of obesity, Church D, with the second highest mean age, was lowest in reports of diabetes, heart disease, hypertension, asthma, and arthritis. Church D also had the lowest report of cancer, although differences across churches were not significant (Table 1).

### Characteristics of kiosk-users

Table 2 shows the results of a multiple logistic regression conducted to obtain independent associations of demographic characteristics and number of health conditions reported to use of the kiosk. All associations are adjusted for church.

**Table 2:** **Association of demographic characteristics, self-report of health conditions, and family member health status by use of faith-based kiosk (all churches) using multiple logistic regression**.

	Kiosk-Use				Total	aOR^c^	95% CI^d^	*p*-Value
	Yes		No	
	*n*^a^	(%)^b^	*n*	(%)				
Age
<40	158	37.6	551	47.8	709	Reference		
40–59.9	196	46.7	429	37.2	625	1.63	(1.27, 2.09)	<0.001
≥60	66	15.7	173	15.0	239	1.52	(1.07, 2.15)	0.019
Race
Non-African-American	47	11.2	139	12.1	186	1.06	(0.74, 1.52)	0.738
African-American	373	88.8	1014	87.9	1387			
Gender
Male	79	20.5	233	22.7	312	1.20	(0.89, 1.60)	0.230
Female	306	79.4	795	77.3	1101			
Self-report of health conditions
None reported	80	21.2	239	23.1	319	Reference		
1 Reported	112	29.6	325	31.4	437	1.07	(0.77, 1.50)	0.688
2 Reported	97	25.7	224	21.7	321	1.43	(1.00, 2.03)	0.050
3 Or more reported	89	23.5	245	23.7	334	1.20	(0.84, 1.72)	0.309
Family member with health condition
No	132	44.3	396	50.4	528	Reference		
Yes	166	55.7	390	49.6	556	1.29	(0.98, 1.69)	0.069

*^a^Number of persons in the specified category*.

*^b^Percentage of the total for that strata; percentage of the total or number of persons in strata/total number with information for this variable*.

*^c^Adjusted odds ratio; all models adjusted for church*.

*^d^95% Confidence interval for adjusted odds ratio*.

Older age was associated with kiosk-use: adjusted odds ratios (OR) and confidence intervals (CI) for “40–59.9 years” and “ ≥60 years” were, OR = 1.63, 95% CI:1.27–2.09) and OR = 1.52, 95% CI:1.07–2.15;(*p* < 0.001 and *p* = 0.019), respectively. Race and gender were not associated with use of the kiosk. Compared to respondents reporting no health conditions, respondents with two health conditions were more likely to use the kiosk (*p* = 0.05). A higher percentage of respondents who reported a family member with a health condition used the kiosk compared to those without such a report, [OR = 1.29, 95% CI:0.98, 1.69], but this was of borderline significance (*p* = 0.069).

We examined the association of gender to sections viewed in the kiosk. Bivariate *p*-values <0.20 for chi-square analysis of section viewed by gender included Diabetes (*p* = 0.016), Power of Prayer (*p* = 0.097), Support and Resources (*p* = 0.026), and High Blood Pressure (*p* = 0.150). Results of multiple logistic regression, adjusting for age and church membership, are shown in Table 3. Men were more likely to view Diabetes [aOR = 1.87 95% CI:1.10–3.17] (*p* = 0.02) and a higher percentage (not significant) viewed High Blood Pressure (53.2% of men vs. 44.1% of women). A higher percentage of women viewed Support and Resources content (13.9% of men vs. 25.8% of women), which was very close to our predetermined level of statistical significance [aOR = 0.49, 95% CI: 0.23–1.04] (*p* = 0.062).

**Table 3:** **Association between gender and kiosk sections viewed using multiple logistic regression (model size = 385)**.

	Male *n* (%)	Female *n* (%)	aOR^a^	95% CI	*p*-Value
**Section(s) viewed**					
Diabetes
No	40 (50.6%)	200 (65.4%)	1.87	(1.10, 3.17)	0.020
Yes	39 (49.4%)	106 (34.6%)			
Power of prayer					
No	62 (78.5%)	211 (69.0%)	0.61	(0.32, 1.18)	0.141
Yes	17 (21.5%)	95 (31.1%)			
Support and resources					
No	68 (86.1%)	227 (74.2%)	0.49	(0.23, 1.04)	0.062
Yes	11 (13.9%)	79 (25.8%)			
High blood pressure					
No	37 (46.8%)	171 (55.9%)	1.39	(0.80, 2.43)	0.243
Yes	42 (53.2%)	135 (44.1%)			

*^a^Adjusted odds ratio and 95% Confidence Interval; all models adjusted for church and age*.

### Self-report of behavior change

Report of behavior change ranged from 69% for increased physical activity to 5.5% for receipt of a flu shot. In addition to increases in physical activity, 68.8% reported changes in diet, and 65.5 reported better communication with a physician. A total of 14.5% reported no change in behavior after viewing the kiosk.

Table 4 shows the results of the multivariable analysis for association of selected variables to self-report of behavior change. Adjusted OR were elevated for African-American race, male gender, and report of a health condition. The latter relationship reached statistical significance only for persons reporting one health condition. Also significant was behavior change reported among kiosk-users caring for a family member with a health condition (Table 4).

**Table 4:** **Factors associated with self-report of any change(s) in health behavior among kiosk-users using multiple logistic regression**.

	aOR^a^	95% CI^b^	*p*-Value
Age
<40	Reference		
40–59.9	0.94	(0.40–2.18)	0.877
≥60	0.86	(0.25–2.95)	0.805
African-American race	1.80	(0.38–8.47)	0.457
Male gender	1.78	(0.63–5.05)	0.279
Self-report of health conditions
None reported	Reference		
1 Reported	3.47	(1.11–10.87)	0.033
2 Reported	1.89	(0.66–5.42)	0.234
3 Or more reported	1.27	(0.45–3.59)	0.652
Family member with a health condition	3.56	(1.39–9.10)	0.008

*^a^Adjusted odds ratio; overall model adjusted for church*.

*^b^95% Confidence interval for adjusted odds ratio*.

## Discussion

We introduced a Faith-based kiosk to four churches with predominantly African-American congregations and collected descriptive data on users. The kiosk focused on a variety of health areas and incorporated religious principles that corroborated healthy living. The kiosk also was a source of information on community resources.

The main findings of our analysis apply to the use of the kiosk. Across the churches, approximately 25% of the survey respondents reported using the kiosk and more than half who used the kiosk for a specific health condition reported making positive changes in health-related behaviors. Congregant characteristics associated with kiosk-use included age (persons 40+ were more likely to use the kiosk) and having a health condition (e.g., diabetes, hypertension, etc.). As might be expected, having a health condition, and/or caring for a family member with a health condition was associated with self-report of change; however, these factors are likely what attracted congregants to the kiosk. Our results also suggest that men used the kiosk more to obtain information; whereas women used the kiosk to look for supportive resources. The literature suggests that African-American men are less likely to visit the physician and less likely to ask questions during a clinic visit. A kiosk may represent a more acceptable vehicle for delivering health information to specific subgroups, such as African-American men. According to our survey, men were as likely as women to use the kiosk, despite selecting different content, and results of this survey could be used to develop gender-specific promotion messages to encourage use.

As might be expected, having a health condition and/or caring for a family member with a health condition was associated with self-report of change. These factors are likely what attracted congregants to the kiosk.

Our results also illustrate how the “collective health” of congregations can vary. Interesting differences in congregant characteristics were noted across churches. Older congregations reported more health problems, with the important exception of obesity, for which the prevalence did not differ significantly between churches, regardless of age or gender distribution. Church D, with the second highest mean age, actually had a lower proportion of respondents with diabetes, heart disease, high blood pressure, asthma, arthritis, and cancer. Possible reasons for this difference include (1) the mean age of Church D is only indicative of survey respondents and not the entire church congregation; (2) healthier respondents were more likely to complete the survey at Church D; (3) healthier persons are drawn to healthier congregations; or (4) these differences are related to variations in socioeconomic status between churches. Church D also had the largest percentage of congregants who reported using the kiosk. We posit that the above trend could also be due to church leadership or a church environment that is supportive of health improvements and a healthy lifestyle. Reasons for variations in collective health across congregations should be explored as this may inform church-based interventions.

To our knowledge, there are few faith-based kiosk programs aimed at promoting healthy behaviors in use in urban, African-American churches. Health based kiosks are present in a variety of community settings but publications are few. Some findings from our study are consistent with earlier research regarding health kiosks, such as users being older than non-users and women more likely to be users than men (16, 17). In considering earlier studies, our report is unique in at least two ways: (1) the majority of the work in this area has been conducted outside of the United States, and did not include respondents from ethnic populations; and (2) a noticeably higher percentage of respondents reporting use of the Faith-based kiosk (26.7%) compared to prior reports in the literature of 13–16% (16–18).

There are several limitations to our report. Response rates were generally 13–15% as best could be estimated using church statistics on the number of members on the roster at each church, and ranged from 46 to 84% using estimates for the number of congregants attending services on any given Sunday. Our estimates for self-report of health conditions should not be viewed as a true prevalence since our sample was not selected randomly. Sicker respondents are less likely to attend church, and therefore, may be underrepresented in our sample. As it stands, our results apply only to those congregants present and choosing to respond to the survey. We do not have information on self-report of behavior change for non-kiosk-users because of skip patterns in the questionnaire. Finally, we do not have accurate, objective measures of SES for survey respondents or congregations.

Using a kiosk to deliver health information has many challenges. In addition to the cost and installation of the kiosk, the number of kiosks necessary to serve a large congregation of 1000–2000 persons must be considered. Funding and technical expertise is required to download and analyze data, and to cover on or offsite technical support. Notwithstanding these challenges, a faith-based kiosk is an excellent way to deliver health education, provide information on available church or community resources, and reinforce health messages in the context of spiritual beliefs. Simultaneously, kiosks could be programed to obtain information on the health status of users, allowing compilation of data for aggregate reports, i.e., a church “Report Card,” which could rally churches to monitor and adopt better health behaviors as a congregation, providing an opportunity for targeted health promotion.

Churches might be viewed as self-contained communities, or portals into a given community, through which a sizeable number of individuals can be reached. In addition to the data that we have collected, collection of detailed characteristics of church leadership and “environment” (i.e., the prioritization of health programs among other existing programs) will also provide valuable information on how congregations as a whole can be motivated to change behavior. Results of this descriptive analysis can inform the feasibility, acceptability, and the potential for Faith-based kiosks to promote health in urban and underserved communities. Next steps include a more rigorous evaluation that includes a comparison group and more objective and clinical measurement of behavioral and health outcomes.

## Conflict of Interest Statement

The authors declare that the research was conducted in the absence of any commercial or financial relationships that could be construed as a potential conflict of interest.
